# A Study on Image Quality in Polarization-Resolved Second Harmonic Generation Microscopy

**DOI:** 10.1038/s41598-017-15257-0

**Published:** 2017-11-13

**Authors:** Stefan G. Stanciu, Francisco J. Ávila, Radu Hristu, Juan M. Bueno

**Affiliations:** 1 Center for Microscopy-Microanalysis and Information Processing, University Politehnica of Bucharest, Bucharest, Romania; 2 Laboratorio de Óptica, Universidad de Murcia, Murcia, Spain

## Abstract

Second harmonic generation (SHG) microscopy represents a very powerful tool for tissue characterization. Polarization-resolved SHG (PSHG) microscopy extends the potential of SHG, by exploiting the dependence of SHG signals on the polarization state of the excitation beam. Among others, this dependence translates to the fact that SHG images collected under different polarization configurations exhibit distinct characteristics in terms of content and appearance. These characteristics hold deep implications over image quality, as perceived by human observers or by image analysis methods custom designed to automatically extract a quality factor from digital images. Our work addresses this subject, by investigating how basic image properties and the outputs of no-reference image quality assessment methods correlate to human expert opinion in the case of PSHG micrographs. Our evaluation framework is based on SHG imaging of collagen-based ocular tissues under different linear and elliptical polarization states of the incident light.

## Introduction

Second Harmonic Generation (SHG) microscopy is regarded nowadays as a very useful and powerful tool for characterizing biological tissues^1,2^. Its potential originates from the ability to image in a label-free manner non-centrosymmetric structures, which exhibit a non-vanishing second-order susceptibility tensor χ^(2)^. Under the influence of an external electric field, such structures generate a nonlinear optical signal at exactly half the wavelength of the excitation source. This emission can be easily isolated from the excitation wavelength or from associated fluorescence signals by using spectral filters. Most SHG applications that focus on physiological assessment and disease diagnostics rely on imaging type-I collagen^3–6^, the most abundant protein in the human body and the main structural protein in the extracellular matrix of animal tissues. The investigation of collagen distribution in tissues with SHG enables a precise and non-invasive assessment of extracellular matrix modifications, which represent a hallmark for a wide range of pathologies, including cancer^7,8^.

Polarization-resolved SHG (PSHG) microscopy extends the potential of SHG, by exploiting the fact that this nonlinear signal is sensitive to the polarization state of the excitation beam^9^. In the case of collagen-based tissues, the SHG emission depends on the alignment between the collagen fibers/fibrils and the polarization of the excitation light^10–12^. The additional dimension available in PSHG data sets can be used to analyze the optical anisotropy and hence to better probe the molecular organization and the external arrangement of collagen-based structures^10–16^. With the hierarchical organization of collagen being closely intertwined with the biophysical, mechanical and hence functional properties of most tissues, PSHG’s potential can be steered towards finding answers to important questions over diseases genesis, progression and treatment. Moreover, PSHG can potentially be used as a non-invasive tool for *in-vivo* diagnostics, replacing in certain scenarios the need for traditional histopathologic approaches based on excisional biopsies and tissue staining.

Based on the mechanisms above discussed, imaging a specific area of a collagenous tissue with PSHG under different polarization configurations can result in a set of SHG images, each of these having distinct characteristics in terms of content and appearance. The information available in such PSHG sets can be exploited to extract quantitative measures with specific physiological or pathological relevance^13,14,16–19^. In the same time, PSHG data sets can be also subjected to qualitative analyses performed by either human or automated experts. In this latter case, a unique single image from an entire PSHG set is typically selected to represent the observed scene. The process of selecting this single image that best represents the observed scene can be difficult and time demanding. Due to this, the choice is usually based on pixel intensity criteria. In such approaches, image brightness is routinely used as a selection criterion, and hence the image exhibiting highest average pixel intensity is considered. At this point an important question naturally arises with respect to this subjective selection procedure: Does the brightest image corresponds to the one with the best quality? Answering this question is not easy, since the definition of “image quality” is not only subjective but it is also very application-dependent. In this work we try to shed more light in this direction. We investigate the effects of different polarization states of the incident light on the quality of PSHG images, as perceived by human experts and automated image quality assessment methods. Special emphasis is placed on identifying which of the methods developed in this purpose over the past years by the digital image processing community are best aligned to human expert opinion in the case of PSHG micrographs collected under various polarization states of the incident light. The samples used as support in this experiment consist in a number of collagen-based ocular tissues; these specimens were chosen because of their relevance with respect to potential pre-clinical/clinical implementations of the herein approach. Although SHG (and PSHG) imaging is possible in both forward and backward configurations^20,21^, for similar reasons a backscattered PSHG geometry was considered, as presented in the Methods section.

In the performed experiments two sets of polarization states are considered: linear (covering the equatorial plane of the Poincaré sphere) and elliptical (located along the vertical meridian), as described in the Methods section. Theoretical aspects related to the importance of linear and elliptical polarization states with respect to type-I collagen SHG imaging are discussed in^22^ and^23^ respectively. In the proposed framework, image quality of PSHG data sets is evaluated in terms of (1) mean-opinion scores (MOS) of human experts, (2) basic image properties such as Average Intensity (brightness), Contrast, Variance or Entropy and (3) by means of automated No-Reference Image Quality Assessment (NR-IQA) methods. The main focus of attention is placed on investigating how (2) and (3) correlate to (1), assessing this aspect by means of prediction accuracy and prediction monotonicity.

Understanding in more detail the relationships that take place between the polarization state of the excitation light, collagen organization and image quality has the potential to enable the development of optimized PSHG image acquisition, processing and analysis protocols, novel adaptive optics strategies and associated image fusion methods.

### Image quality assessment

Image quality assessment (IQA) has always attracted considerable interest, but over the past three decades it became a key topic of concern. The reason is that digital images became broadly available to the general public and started to be acquired, compressed, transmitted, restored, and edited on a routine basis. Nowadays IQA methods play an important role in the design and benchmarking of imaging devices, and represent the necessary tools to evaluate up to what degree an image is degraded by various distortions and operations to which it is subjected.

Current IQA methodologies are split in two main categories: subjective and objective approaches. While the former are based on the quality scores provided by human experts, the latter rely on mathematical models that can automatically provide an estimate over the perceived image quality (which is consistent with that of a human observer). These objective methods are also divided into three main classes according to the availability of a distortion-free reference image: (i) NR-IQA, a.k.a. “blind”, (ii) Reduced-Reference IQA and (iii) Full-Reference IQA (FR-IQA).

FR-IQA methods yield a prediction of the visual quality of a target image, relative to the reference image, which is considered to be of optimal quality^24^. The use of these FR-IQA approaches in the realm of microscopy is difficult, due to the typical unavailability of reference images. On the opposite, NR-IQA methods predict the image quality based solely on the information contained in the tested image, thus their use in association with microscopy images is straightforward. On the other hand, one should consider that the great majority of consecrated NR-IQA methods, e.g.^25–29^, have been designed taking into account the characteristics and specifics of natural images collected with digital cameras, whereas images collected by microscopy systems differ due to the nature of the imaged scenes and the acquisition mechanisms. This suggests that the application of such NR-IQA metrics to microscopy data sets may lead to unpredictable results. Although a number of microscopy oriented NR-IQA approaches have been reported^30–34^, these were mainly developed to address very specific applications, what might generate similar concerns over their reliability and predictability when used in other scenarios. To the best of our knowledge, the use of NR-IQA methods in combination with PSHG data sets represents a subject that has not been previously addressed.

Our work has been focused on investigating some of the effects on image quality of the polarization states typically available in a PSHG system and typically used for collagen tissue imaging. While in most PSHG experiments image quality is mainly considered in terms of the image intensity (brightness), our experiment extends this approach by adding to the evaluation framework additional basic metrics such as Contrast, Variance or Entropy (defined in the Methods section). Furthermore, we additionally employ 15 prominent NR-IQA methods developed by the image processing community: BRISQUE^25^, BLIINDS2^26^, SSEQ^35^, BIQAA^36^, BIQI^28^, CPBD^37^, BIBLE^38^, CDIQA^39^, DCTSP^40^, MLV^41^, NIQE^27^, QAC^42^, SML^43^, SDQI^44^, ILNIQE^45^. The evaluation framework includes as well a simplistic quality estimator called ARDE^31^, previously designed taking into account typical image properties considered by human experts when they investigate laser scanning microscopy images. A discussion over the mechanisms of these NR-IQA methods (16 in total here) falls outside the scope of our paper, but the interested readers can find detailed information in the original publications and source codes. Complete algorithm titles are provided in the Methods section.

## Results

### Evaluation Framework

For every sample here used 24 PSHG images were involved in the study, 12 corresponding to the considered Linear Polarization States (LPS), and 12 corresponding to the considered Elliptical Polarization States (EPS); more information is provided in the Methods section. Based on the image sets corresponding to LPS and EPS, a third image set considering all polarization states (APS) has been assembled for IQA testing purposes. Since LPS and EPS image sets contain duplicates for the cases where the vertical meridian and the equatorial planes of the Poincaré sphere intersect (i.e. L_0 and E_0; L_90 and E_90, see experimental configuration figure in the Methods section), to avoid redundancy images E_0 and E_90 were removed from the APS image set. For rationales linked to conciseness, in the following sub-sections we restrict to presenting the results obtained for the APS image sets.

In order to gain insights over how the considered image properties and NR-IQA methods align to the opinions of human experts, for each of the considered image sets we perform an in-depth analysis using a consecrated set of correlation measures: (1) the Pearson Linear Correlation Coefficient (PLCC), (2) the Spearman Rank-Order Correlation Coefficient (SROCC), and (3) the Root Mean Square Error (RMSE) between the *predicted MOS* and the *actual MOS* provided by human experts. By *predicted MOS*, we refer to the basic image properties described in the Methods section, and to the outputs of the 16 considered NR-IQA methods. By *actual MOS*, we refer to the scores assigned by human experts to the evaluated PSHG images on a scale ranging from 1 (worst) to 5 (best) in steps of 0.5. The human experts independently scored the PSHG images of the evaluated sets, without apriori knowledge on the scoring of others. The scoring was done based on criteria such as contrast, ratio of bright details over dark background, dynamic range exploitation, sharpness and visibility of features of interest. All of these aspects have been assessed in a subjective manner, without employing any specialized software or mathematical models.

In terms of correlation coefficients, the SROCC indicates the prediction monotonicity, whereas PLCC and RMSE serve as measures of prediction accuracy^46^. A better correlation of the NR-IQA metrics with the perception of human experts (MOS), means a value close to one for PLCC and SROCC and a value close to zero for RMSE^25,29,35,38,47^. Details on the non-linear mapping of predicted MOS to actual MOS, prior to computing PLCC, SROCC and RMSE values are also provided in the Methods section.

### Correlation measures: Human Expert Opinion vs. Basic Image Properties

In this section, we evaluate how the considered basic image properties correlate to the opinions of human experts for the five investigated biological specimens (presented in the Methods section). Tables 1, 2 and 3 present the PLCC, SROCC and RMSE coefficients between the MOS of actual human experts and the numerical values associated to the considered basic image metrics: Average Intensity, Contrast-per-pixel^48^, Variance and Entropy. On average it can be said that among the four evaluated image properties, Average Intensity is best correlated to human expert opinion, while Entropy is second. However, this does not apply in the case of all image sets, see for example the case of the sets corresponding to samples #1 or #2, where Contrast-per-pixel is better correlated to MOS than both.

**Table 1:** PLCC of the considered image properties across the five tested PSHG image sets.

	Sample #1	Sample #2	Sample #3	Sample #4	Sample #5	Mean
Average Intensity	0.4124	0.6337	0.9504	0.9826	0.6357	0.7230
Entropy	0.3189	0.2862	0.7713	0.9295	0.7074	0.6027
Contrast-per-pixel	0.7739	0.6444	0.6967	0.0394	0.1553	0.4619
Variance	0.1227	0.2176	0.1453	0.9302	0.4793	0.3790

**Table 2:** SROCC of the considered image properties across the five tested PSHG image sets.

	Sample #1	Sample #2	Sample #3	Sample #4	Sample #5	Mean
Average Intensity	0.2761	0.5576	0.9243	0.9762	0.6078	0.6684
Entropy	0.4777	0.0926	0.8227	0.9450	0.6299	0.5936
Contrast-per-pixel	0.8038	0.7053	0.6048	0.0976	0.1813	0.4786
Variance	0.2176	0.1051	0.0119	0.9694	0.5591	0.3726

**Table 3:** RMSE of the considered image properties across the five tested PSHG image sets.

	Sample #1	Sample #2	Sample #3	Sample #4	Sample #5	Mean
Average Intensity	0.8383	0.6942	0.4050	0.2383	1.0629	0.6477
Entropy	0.8722	0.8596	0.8291	0.4730	0.9624	0.7993
Variance	0.9133	0.8756	1.2888	0.4709	1.1923	0.9482
Contrast-per-pixel	0.5827	0.6860	0.9345	1.2818	1.3422	0.9654

### Correlation measures: Human Expert Opinion vs. NR-IQA methods

In this section, we evaluate how the 16 considered NR-IQA methods correlate to the opinions of human experts, and how they compare to the Average Intensity. The values of PLCC, SROCC and RMSE correlation measures for the considered NR-IQA methods are shown in Tables 4, 5 and 6. For a direct visualization, the NR-IQA techniques that better predict the opinion of human experts compared to the Average Intensity are listed in italicized bold font.

**Table 4:** PLCC of the considered NR-IQA methods across the five tested PSHG image sets.

	Sample #1	Sample #2	Sample #3	Sample #4	Sample #5	Mean
***SDQI***	0.8909	0.9188	0.9154	0.9691	0.7928	0.8974
***DCTSP***	0.8991	0.6404	0.9365	0.9513	0.8664	0.8587
***CPBD***	0.8838	0.6370	0.9345	0.9283	0.7501	0.8267
***BIBLE***	0.8023	0.6968	0.9339	0.9400	0.7075	0.8161
***SSEQ***	0.8328	0.6027	0.9594	0.9182	0.5836	0.7793
***BIQAA***	0.6848	0.4887	0.8955	0.8702	0.8789	0.7636
***ARDE***	0.6616	0.4191	0.7580	0.9824	0.8022	0.7247
MLV	0.7926	0.6329	0.8418	0.7317	0.5815	0.7161
BLIINDS2	0.4153	0.6505	0.5389	0.9522	0.8699	0.6854
BIQI	0.5680	0.4724	0.9389	0.8035	0.3957	0.6357
SML	0.7900	0.6529	0.7758	0.3183	0.4619	0.5998
QAC	0.4292	0.6902	0.8642	0.3085	0.5254	0.5635
CDIQA	0.4372	0.2846	0.4696	0.9755	0.7200	0.5774
NIQE	0.8348	0.3951	0.0683	0.6359	0.6524	0.5173
BRISQUE	0.8567	0.7474	0.3515	0.0691	0.5032	0.5056
ILNIQE	0.5516	0.5495	0.4487	0.5639	0.1519	0.4531

**Table 5:** SROCC of the considered NR-IQA methods across the five tested PSHG image sets.

	Sample #1	Sample #2	Sample #3	Sample #4	Sample #5	Mean
***SDQI***	0.7918	0.8946	0.8965	0.9410	0.8043	0.8656
***DCTSP***	0.8316	0.5900	0.8653	0.9240	0.8621	0.8146
***CPBD***	0.8265	0.6741	0.8613	0.9087	0.7403	0.8022
***ARDE***	0.6714	0.4808	0.8880	0.9756	0.7681	0.7568
***BIBLE***	0.6260	0.7229	0.8482	0.8690	0.6780	0.7488
***SSEQ***	0.7152	0.6235	0.9180	0.8179	0.5285	0.7206
***BIQAA***	0.7328	0.4581	0.8760	0.7102	0.8088	0.7172
***MLV***	0.7668	0.6729	0.7609	0.6597	0.6185	0.6958
***BLIINDS2***	0.5226	0.5614	0.5177	0.9421	0.8243	0.6736
SML	0.8117	0.7195	0.7126	0.2972	0.4820	0.6046
BIQI	0.5726	0.5246	0.8426	0.6665	0.4112	0.6035
CDIQA	0.5357	0.0750	0.6116	0.9541	0.6372	0.5627
QAC	0.4692	0.7212	0.7762	0.3023	0.4123	0.5362
NIQE	0.7350	0.3711	0.0295	0.5888	0.6973	0.4843
ILNIQE	0.4942	0.5610	0.4335	0.5706	0.1711	0.4461
BRISQUE	0.8339	0.7860	0.1305	0.0703	0.3999	0.4441

**Table 6:** RMSE of the considered NR-IQA methods across the five tested PSHG image sets.

	Sample #1	Sample #2	Sample #3	Sample #4	Sample #5	Mean
***SDQI***	0.4180	0.3541	0.5244	0.3162	0.8280	0.4881
***DCTSP***	0.4028	0.6890	0.4568	0.3956	0.6782	0.5245
***CPBD***	0.4305	0.6916	0.4638	0.4768	0.8983	0.5922
***BIBLE***	0.5492	0.6435	0.4659	0.4375	0.9600	0.6112
***SSEQ***	0.5094	0.7158	0.3673	0.5082	1.1031	0.6408
BIQAA	0.6706	0.7827	0.5798	0.6321	0.6480	0.6626
ARDE	0.6900	0.8145	0.8496	0.2394	0.8110	0.6809
BLIINDS2	0.8371	0.6813	1.0973	0.3921	0.6700	0.7356
MLV	0.5611	0.6946	0.7031	0.8744	1.1051	0.7877
BIQI	0.7574	0.7981	0.4482	0.7637	1.2484	0.8032
CDIQA	0.8276	0.8600	1.1500	0.2821	0.9427	0.8125
QAC	0.8312	0.6499	0.6554	1.2202	1.1559	0.9025
SML	0.5642	0.6795	0.8219	1.2161	1.2988	0.9161
NIQE	0.5067	0.8241	1.2996	0.9900	1.0295	0.9300
BRISQUE	0.4747	0.5960	1.2195	1.2797	1.1812	0.9502
ILNIQE	0.7678	0.7495	1.1730	1.0600	1.3427	1.0186

From the prediction accuracy perspective (see Tables 4 and 6): 7 out of 16 and 5 out of 16 NR-IQA methods outperform Average Intensity according to PLCC and RMSE analyses respectively. According to both PLCC and RMSE scores, SDQI, DCTSP, CPBD and BIBLE are best in terms of prediction accuracy. It is interesting to notice that 3 out of these 4 techniques, (namely DCTSP, CPBD and BIBLE) are regarded by their authors as methods for blur/sharpness detection methods rather than image quality estimators of general use. This enforces the idea that image sharpness has a very significant importance for human experts when they evaluate the quality of PSHG images.

From the prediction monotonicity perspective (see Table 5): 8 out of 16 NR-IQA methods provide better results than the Average Intensity according to the SROCC analysis. SDQI, DCTSP and CPBD occupy again the top three positions, while BIBLE drops from the fourth position to the fifth, the fourth position being occupied by ARDE. It is interesting to note that ARDE is a basic image quality estimator that has been designed taking into account typical image properties that human experts consider when they investigate laser scanning microscopy images^31^. ARDE provides also better prediction accuracy than the average image intensity according to the PLCC analysis (Table 4).

### Visual Perspective over Image Quality in PSHG data sets

**Figure 1:**
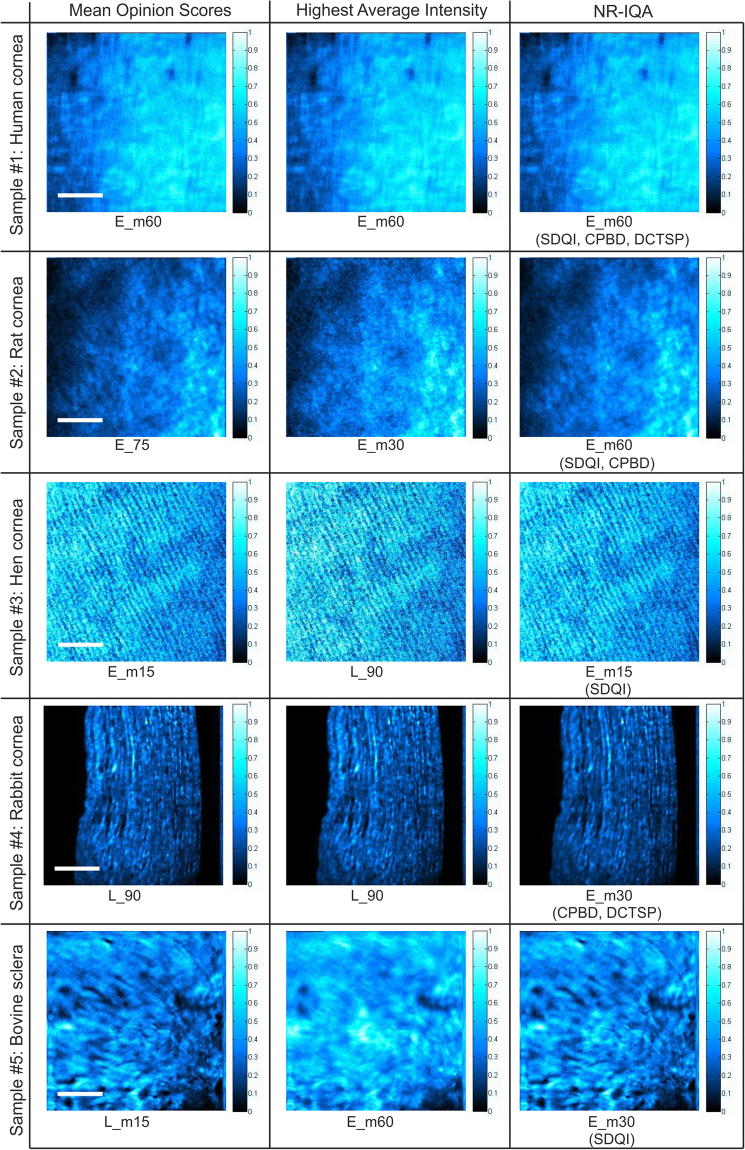
PSGH image instances with highest MOS, highest Average Intensity and most voted by the top three NR-IQA methods, SDQI, DCTSP and CPBD (see text for more details on the voting scheme). The signal scale bar is shown at the right of the normalized SHG images. Scale bar: 50 µm.

For a better understanding of the results reported in the previous sections, a visual perspective is provided in Fig. 1 for every APS image set. The PSHG image perceived as best by the human experts (i.e. MOS) is shown on the left column. PSHG images of the central column correspond to those with the highest Average Intensity (i.e. the brightest one). Finally, the panels on the right are those images selected as best in a “majority voting scheme” by the top three NR-IQA methods according to the PLCC, SROCC and RMSE analyses. In this voting scheme SDQI, DCTSP and CPBD assign their vote to the image in the APS set which scores higher than the rest. The image displayed is the instance in the APS set which obtains the highest number of votes. In the case of image sets of samples #3 and #5, each of the three considered NR-IQA metrics vote different images as best, and in these cases we display in Fig. 1 the image voted by SDQI, the best NR-IQA metrics according to performed correlation analyses. For each image selected based on this voting strategy the names of the responsible NR-IQA metrics are displayed underneath the image instance. The nomenclature of the images in the APS sets is discussed in the Methods section, and the coordinates of the displayed instances can be visualized in Poincaré sphere representations (see Supplementary Fig. S1).

In the scene imaged for generating the Sample #1 set collagen fibers arranged in a cross-hatched pattern can be observed (both horizontal and vertical orientations). In this set the E_m60 instance is the brightest image, the image with highest MOS, and unanimously voted by the top three NR-IQA methods. This fact is of interest since clinical ophthalmologic applications are mainly oriented to human patients where the diagnoses of some pathologies is classically based on the visualization of certain features and the observation of particular changes in the collagen structure.

For samples #2 and #5, the brightest, highest MOS, and NR-IQA voted images are different. This must be due to the fact that a random collagen arrangement is present. Since the collagen fibers exhibit a lack of dominant orientation, different incident polarization states might provide images with similar characteristics. In both sets the NR-IQA voted image is better in terms of information content than the brightest image (according to human expert opinion). In the PSHG images of the rat cornea (sample #2) we can observe sets of fibers with a non-regular distribution, where the presence of undulations is dominant. In the PSHG images of the bovine sclera (sample #5), short and random distributed collagen fibers (typical to the structure of the sclera) can be easily visualized.

For sample #3, the MOS and NR-IQA voted for the same instances. The MOS/NR-IQA most voted instance, E_m15, is less bright than L_90, but details of interest can be better observed as they lack the illumination gradient present in L_90, which biases visual inspection. The content of the PSHG images collected for this hen cornea specimen depicts a stroma structure composed of well-aligned fibers, where both individual thickness and inter-fiber space can easily be computed. In potential diagnostics scenarios, modifications in the value of related parameters can be associated to an edematous pathological process.

For sample #4 the brightest and highest MOS images coincide (L_90). It is worth to mention that the incident polarization for this image is parallel to the organization of the fibers there shown, resulting in maximal SHG intensity. CPBD and DCTSP indicate a different image as the best, however SDQI also provides L_90 as the highest scored image. In these images collected on a histological section of a rabbit cornea specimen the different layers of the corneal stroma can be distinguished. It can be observed that each layer lies parallel to surface of the cornea.

Interestingly, for the five image sets here analyzed the proposed NR-IQA voting scheme always selects as best an image collected under elliptical polarization, independently of the collagen distribution of the samples. This may be connected to the fact that in most cases images collected under elliptical polarization states contain a surplus of information in comparison to the ones collected in a linear polarization configuration^49^. This additional information typically translates in additional image content (e.g. structures, edges), and hence an increased response to operators based on image gradients that provide information over sharpness/focus.

## Discussion

A typical approach in PSHG experiments is to select the brightest image of a set to represent the observed scene for either visual inspection purposes or for subjecting it to automated computer vision methods (e.g. segmentation, object recognition, image classification, etc.). This approach is mainly connected to the fact that in PSHG imaging exists a clear connection between the image intensity and the polarization direction of the excitation beam. This relationship, thoroughly discussed to date^10–12,50,51^, is related to the fact that for tissue areas with collagen fibers exhibiting a preferential orientation the brightest image is achieved when this orientation is matched with a particular polarization configuration. While this phenomena is very important, as it enables a wide variety of numerical methods to quantify collagen organization, e.g.^13,14,17–19^, our experiment indicates that the brightest PSHG image in a set coincides only in particular scenarios with the image perceived as best by human experts. This can be easily observed in Fig. 1, and the results of the performed PLCC, SROCC and RMSE analyses consolidate this claim. In Tables 1, 2 and 3 it can be observed that although the image intensity is on average better aligned to human perception when compared to other evaluated image properties (such as Contrast, Variance or Entropy), the corresponding PLCC, SROCC and RMSE scores are low for some of the tested image sets suggesting a weak correlation between human expert opinion and image brightness.

These initial observations led us to seek alternative ways of ranking PSHG image sets in terms of image quality. In this regard, we have turned our attention to a set of prominent NR-IQA methods. These have been mainly designed taking into account the characteristics of natural images (except ARDE) currently acquired on a daily basis by the general public which nowadays has large-scale access to digital cameras. The PLCC, SROCC and RMSE correlation analyses performed in this second part of the experiment indicate a series of alternatives to image brightness with respect to the problem of estimating the quality of a PSHG image. These alternatives consist on NR-IQA methods that provide better prediction accuracy and prediction monotonicity to the opinions of human experts in terms of PSHG image quality.

Our experiments have been motivated as well by the fact that the great majority of IQA methods reported to date are developed taking into account the characteristics of natural images, whereas images collected by laser scanning microscopy differ due to the nature of the imaged scenes and the acquisition mechanisms. In these circumstances, randomly selecting a NR-IQA method from the literature and applying it to PSHG (or other laser scanning microscopy) image sets can lead to unpredictable results. Shedding more light over which IQA methods are better aligned to microscopy oriented applications is thus very important in our opinion. Studies on this topic are scarce in the literature, making it poorly documented to date despite the huge importance that image quality assessment holds with respect to microscopy imaging. Manually searching for representative images in large-scale image sets collected over a scene of interest is time demanding and subjective; these aspects can be overpassed by employing automated IQA methods. Furthermore, IQA methods hold as well considerable potential for image fusion and scene representation frameworks^52–55^ or adaptive optics^56^ applications, where the quality of the final result is closely related to the performance of the decision criteria that are used. Moreover, appropriate IQA methods could speed-up and optimize the outputs of machine intelligence methods aimed at tissue classification by automatically selecting a single instance from an extended image set of the same scene, which is better suited then others with respect to a specific computer vision methodology, e.g. Bag-of-Features^19^, Deep-Learning^57^. An example in this regard can be found in^58^ where the ARDE^31^ operator was used to select particular image instances from z-stacks collected with Two-Photon Excitation Microscopy on rat liver tissue, to be further used in a tissue classification framework.

To conclude, in this work we have investigated how basic image properties and NR-IQA methods compare to the opinions of human experts in the case of PSHG image sets collected on several types of collagenous ocular tissues. Our results show that, on average, image brightness does better in predicting the opinion of human experts in terms of PSHG image quality, compared to other basic image properties such as Contrast, Variance or Entropy. On the other hand, the performed experiments show that solely using the Average Intensity as a decision criterion for image quality assessment is suitable only in particular cases. Part of the NR-IQA methods reported to date can represent better alternatives in this regard, whereas others provide worse performances. Thus, using NR-IQA methods in association with PSHG image sets is not straightforward, and should be done only after careful benchmarking. In the case of our experiments SDQI, DCTSP and CPBD were found to be the top three NR-IQA metrics that outperform the Average Intensity (brightness) in terms of accurately and monotonously predicting the opinion of human experts over PSHG image quality.

## Methods

### Experimental setup

**Figure 2:**
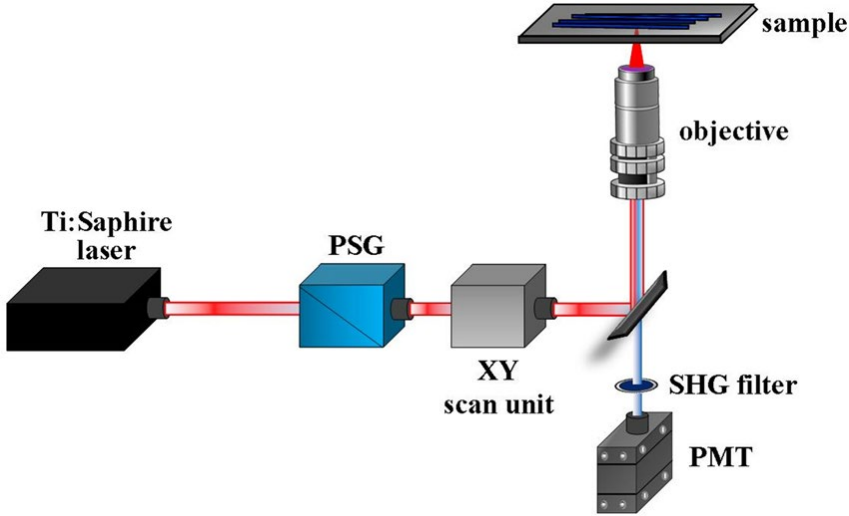
Schematic representation of the custom-built polarimetric SHG microscope used for imaging. See text for further information.

The system used for imaging (Fig. 2) relies on a previously custom-built SHG microscope^59^, which was modified to incorporate a polarization state generator (PSG) into the illumination pathway to modulate the polarization state of the incident light. For illumination, this PSHG system uses a Ti:Shapphire femtosecond laser (120-fs pulses, λ = 800 nm and 76 MHz repetition rate). The excitation beam encounters a XY scanning unit (a pair of non-resonant galvanometric mirrors) after traversing the PSG, and is focused on the sample through a non-immersion objective (20x, NA = 0.5). The backscattered SHG signal emerging from the sample is collected via the same objective and isolated by means of a narrow-band spectral filter (400 ± 10 nm) placed in front of the photomultiplier tube (PMT). The system is fully controlled through a custom Labview^TM^ software. In the case of the presented experiments the average incident laser power ranged between 10 and 50 mW at the sample’s plane.

**Figure 3:**
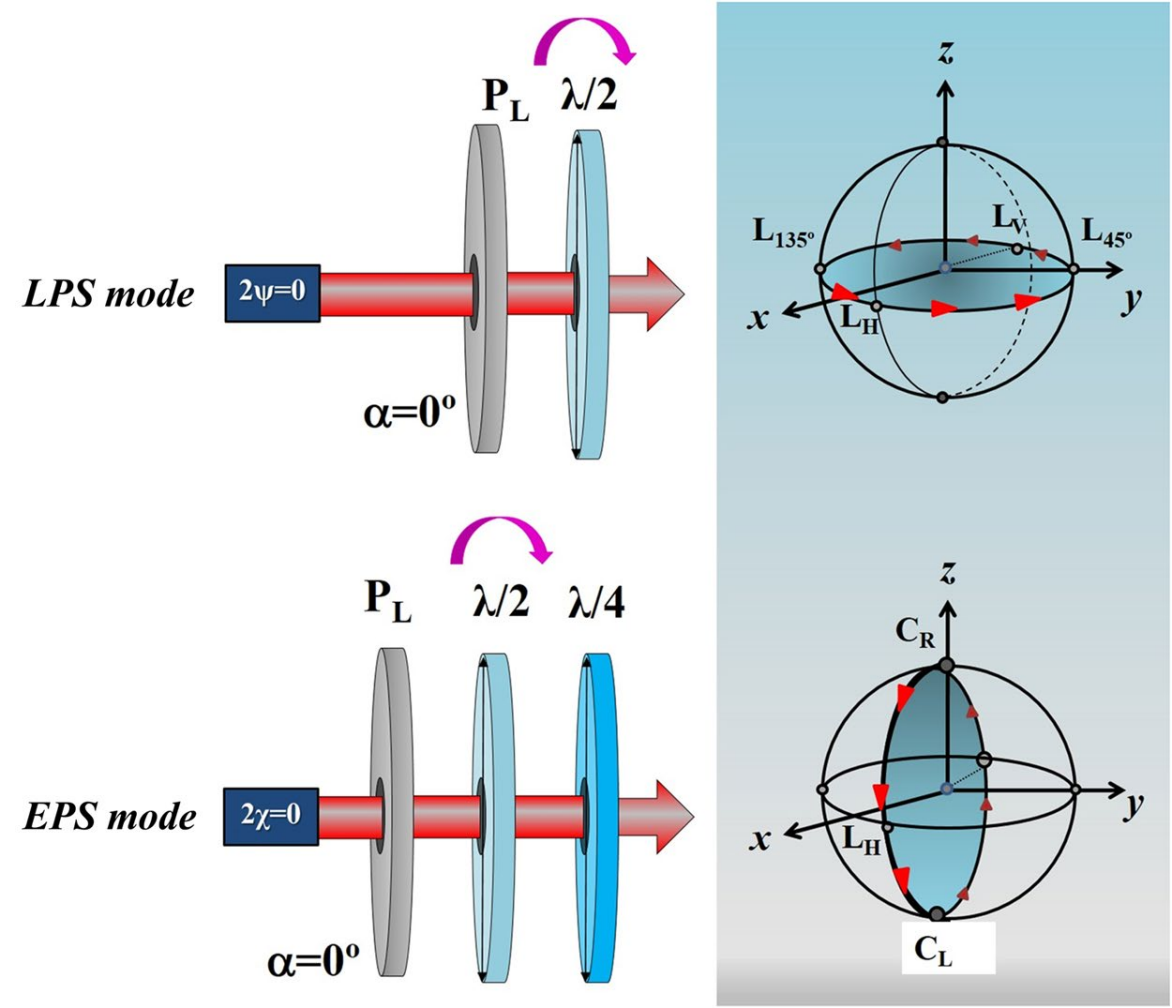
Experimental configuration of the PSG to generate linear and elliptical polarization states. P_L_: linear polarizer; λ/2: rotatory half-wave plate; λ/4: removable quarter-wave plate. The Poincaré spheres on the right show the two sets of polarization states: LPS (on the equatorial plane, upper panel) and EPS (along the vertical meridian, bottom panel).

The PSG incorporated in the experimental setup was designed in a double configuration, to generate sets of linear (null ellipticity, 2ψ = 0) and elliptical polarization states (null azimuth, 2χ = 0) as described in detail in^50^. Figure 3 shows a schematic diagram of the PSG to better understand how these considered polarization states are produced.

A fixed horizontal linear polarizer (P_L_), a rotatory half-wave plate (λ/2) and a removable quarter-wave plate (λ/4) are the three optical components of the PSG. The image sets collected under LPS were obtained by rotating the λ/2, in a P_L_ + λ/2 combination (λ/4 excluded from the light path). These LPS are located 15 deg apart in azimuth on the equatorial plane of the Poincaré sphere. For the combination P_L_ + λ/2 + λ/4 these linear states are switched to a set of elliptical ones (EPS, with 2χ = 0). These are located along the vertical meridian of the Poincaré sphere (including left and right circular) in steps of 15 deg in ellipticity.

When the λ/4 plate is introduced in the optical pathway (EPS configuration) a slight change in the intensity beam occurs due to the additional light absorption corresponding to this optical element. This fact can hold an influence over the intensity of the corresponding SHG images and thus bias the opinions of human experts and the outputs of the considered image quality estimators. This potential issue has been addressed by slightly adjusting the position of a neutral density filter (not displayed in Fig. 1) in order to achieve identical average intensities for the image instances acquired in the positions where the vertical meridian and equatorial plane of the Poincaré sphere intersect.

### Investigated samples

Five non-stained collagen-based ocular tissues (namely cornea and sclera), were involved in the present study. In particular the specimens here used correspond to *ex-vivo* corneas from human (sample #1), rat (sample #2), and adult chicken (sample #3), all them fixed by paraformaldehyde. A histological section of a rabbit cornea (embedded in paraffin) and an *ex-vivo* bovine sclera (fixed by paraformaldehyde) were named as samples #4 and #5 respectively. The importance of SHG and PSHG imaging with respect to ocular tissues has been thoroughly discussed to date in the literature^60–67^.

The use of animal and human tissue samples in this study was approved by the Universidad de Murcia ethics committee and all procedures were carried out in accordance with the approved guidelines, which also regulate the subject of informed consent for samples of human origin.

### Tested image sets (Polarimetric Imaging procedure)

By using the described imaging setup SHG images of the five samples discussed in the Results section were acquired for both sets of polarization, LPS and EPS. For each incident polarization state three individual SHG images (210 × 210 µm^2^) were recorded (frame rate: 1 Hz, image size: 256 × 256 pixels). These were averaged to reduce noise with custom Matlab^TM^ software. Then, for both LPS and EPS configurations 12 SHG images are used. The nomenclature of these images is provided in the following:

- LPS image set: L_χ, where 2χ represent the angle in degrees of the azimuth on the horizontal meridian of the Poincaré sphere (negative χ values are indicated with “m”). The images of this set are: L_0, L_15, L_30, L_45, L_60, L_75, L_90, L_m15, L_m30, L_m45, L_m60 and L_m75 (χ = 0 corresponds to horizontally polarized light; χ = 90 corresponds to vertically polarized light).
- EPS image set: E_φ; where 2φ represent the angle in degrees on the vertical meridian with χ = 0 of the Poincaré sphere (negative φ values are indicated with “m”). The images included in this set are E_0, E_15, E_30, E_45, E_60, E_75, E_90, E_m15, E_m30, E_m45, E_m60 and E_m75 (φ = 45° and −45° correspond to right and left circular polarization respectively).

In terms of the ellipse of polarization, the states of the second set correspond to an ellipse with its axes lying along the horizontal and vertical directions and changing only its ellipticity (but not the azimuth, or slope, of those axes). This means that polarization states E_0 and L_0, and L_90 and E_90 are the same.

### Basic Image Properties of Interest

In Table 7, we provide the mathematical formulas and the significance of the four considered basic image properties.

**Table 7:** Selected list of basic image properties relevant with respect to image quality assessment.

**Basic image metric**	**Equation**
**Average Intensity (M)**: mean value of image pixels, a direct measure of image brightness	$M=\frac{1}{MN}\sum _{i=1}^{M}\sum _{j=1}^{N}{Im}(i,j)\,(1)$
**Contrast-per-pixel**^48^ **(CPP)**: mean difference in grey level between adjacent pixels, a direct measure of deviations in the perceived brightness	$CPP=\frac{1}{MN}\sum _{i=1}^{M}\sum _{j=1}^{N}(\sum _{(m,n\in {R}_{3}^{(i,j)}}Im(i,j)-Im(m,n))\,(2)$
**Variance (VAR)**: average of squared deviation of all pixels from mean, reflects how much the image pixels differ from each other which holds implications for image information quantity	$VAR=\frac{1}{MN}\sum _{i=1}^{M}\sum _{j=1}^{N}{(Im(i,j)-M)}^{2}\,(3)$
**Entropy (E)**: statistical measure of pixel value randomness, can be regarded as a measure of the average information content of an image	$E=\sum _{k=1}^{256}-p(k)\times {\mathrm{log}}_{2}p(k)\,(4)$where *p*(*k*) = the probability of a pixel having intensity *k* and is determined from the normalized 8-bit histogram

### Evaluated NR-IQA algorithms

The evaluated NR-IQA algorithms are presented in Table 8 in chronological order. In the case of 14 of the 16 evaluated algorithms, the source code associated the respective NR-IQA method is provided by the authors either in their publication, or on the group webpage. For BIBLE^38^ the source code was provided by the authors upon request. The ARDE^31^ method was previously developed by the first author (together with two other collaborators). The source code is available upon request.

**Table 8:** Acronyms, titles and year of release of the evaluated NR-IQA.

**Acronym**	***Algorithm name***	***Year of release***
SML^43^	Sum-Modified Laplacian	1994
BIQAA^36^	Blind Image Quality Assessment Through Anisotropy	2007
CPBD^37^	Cumulative Probability Of Blur Detection	2009
BIQI^28^	The Blind Image Quality Index	2010
DCTSP^40^	Discrete Cosine Transform Statistic Prediction Method	2010
ARDE^31^	Automated Reference Detection Estimator	2010
BRISQUE^25^	Blind/Referenceless Image Spatial Quality Evaluator	2012
BLIINDS2^26^	Blind Image Integrity Notator Using Discrete Cosine Transform Statistics	2012
QAC^42^	Blind Image Quality Assessment Based On Quality-Aware Clustering	2013
NIQE^27^	Natural Image Quality Evaluator	2013
SSEQ^35^	Spatial Spectral Entropy Based Quality Index	2014
MLV^41^	Maximum Local Variation For Sharpness Assessment.	2014
ILNIQE^45^	Integrated Local Natural Image Quality Evaluator	2015
CDIQA^39^	No-Reference Quality Metric For Contrast-Distorted Images Based On Natural Scene Statistics	2015
BIBLE^38^	Blind Image Blur Evaluation Using Tchebichef Moments	2016
SDQI^44^	Sparsity Based No-Reference Image Quality Assessment For Automatic Denoising	2017

### Non-linear mapping of predicted MOS to actual MOS

As recommended in^46^, before computing the PLCC, SROCC and RMSE correlation coefficients, a regression function was applied on the Predicted MOS sets in order to provide a nonlinear mapping between these and the actual MOS Scores. For this purpose, similar to^25,29,35,47^ we utilized a logistic function with an added linear term:

5 $$f(x)={\beta }_{1}(\frac{1}{2}-\frac{1}{1+\exp ({\beta }_{2}({x}-{\beta }_{3}))})+{\beta }_{4}x+{\beta }_{5},$$

In Eq. 5, *x* denotes the numerical value given by the considered basic metric (described in Table 7), or the NR-IQA methods, and β_*i* *=* *1*,…,*5*_ are determined by least square fitting to the actual MOS values provided by the human experts.

## Electronic supplementary material

Fig. S1.(PDF 280 kb)
