# Author Correction: A Study on Image Quality in Polarization-Resolved Second Harmonic Generation Microscopy

**DOI:** 10.1038/s41598-018-22949-8

**Published:** 2018-03-13

**Authors:** Stefan G. Stanciu, Francisco J. Ávila, Radu Hristu, Juan M. Bueno

**Affiliations:** 1 Center for Microscopy-Microanalysis and Information Processing, University Politehnica of Bucharest, Bucharest, Romania; 2 Laboratorio de Óptica, Universidad de Murcia, Murcia, Spain

Correction to: *Scientific Reports* 10.1038/s41598-017-15257-0, published online 13 November 2017

This Article contains typographical errors in the Acknowledgements section.

“and the Spanish Society for the Research of Individual Differences (SEIDI), grant FIS2016-76163-R (FJA & JMB)”

should read:

“and the Secretaría de Estado de Investigación, Desarrollo e Innovación (SEIDI), Spain, grant FIS2016-76163-R (JMB & FJA)”

