# Supplementary material for: A Study on Image Quality in Polarization-Resolved Second Harmonic Generation Microscopy
Source: Sci Rep. 2017 Nov 13;7:15476. doi: 10.1038/s41598-017-15257-0 (PMC5684207; doi:10.1038/s41598-017-15257-0)
Supplement: Supplementary file 1 — Fig. S1.(PDF 280 kb) [file 41598_2017_15257_MOESM1_ESM.pdf]

A STUDY ON IMAGE QUALITY IN POLARIZATION-RESOLVED SECOND HARMONIC GENERATION MICROSCOPY

Stefan G. Stanciu[1*], Francisco J. Ávila[2], Radu Hristu[1] and Juan M. Bueno[2*]
[1]Center for Microscopy-Microanalysis and Information Processing, University Politehnica of Bucharest, Romania
[2]Laboratorio de Óptica, Universidad de Murcia, Spain
***E-mails: stefan.stanciu@cmmip-upb.org; bueno@um.es**

Supplementary Information

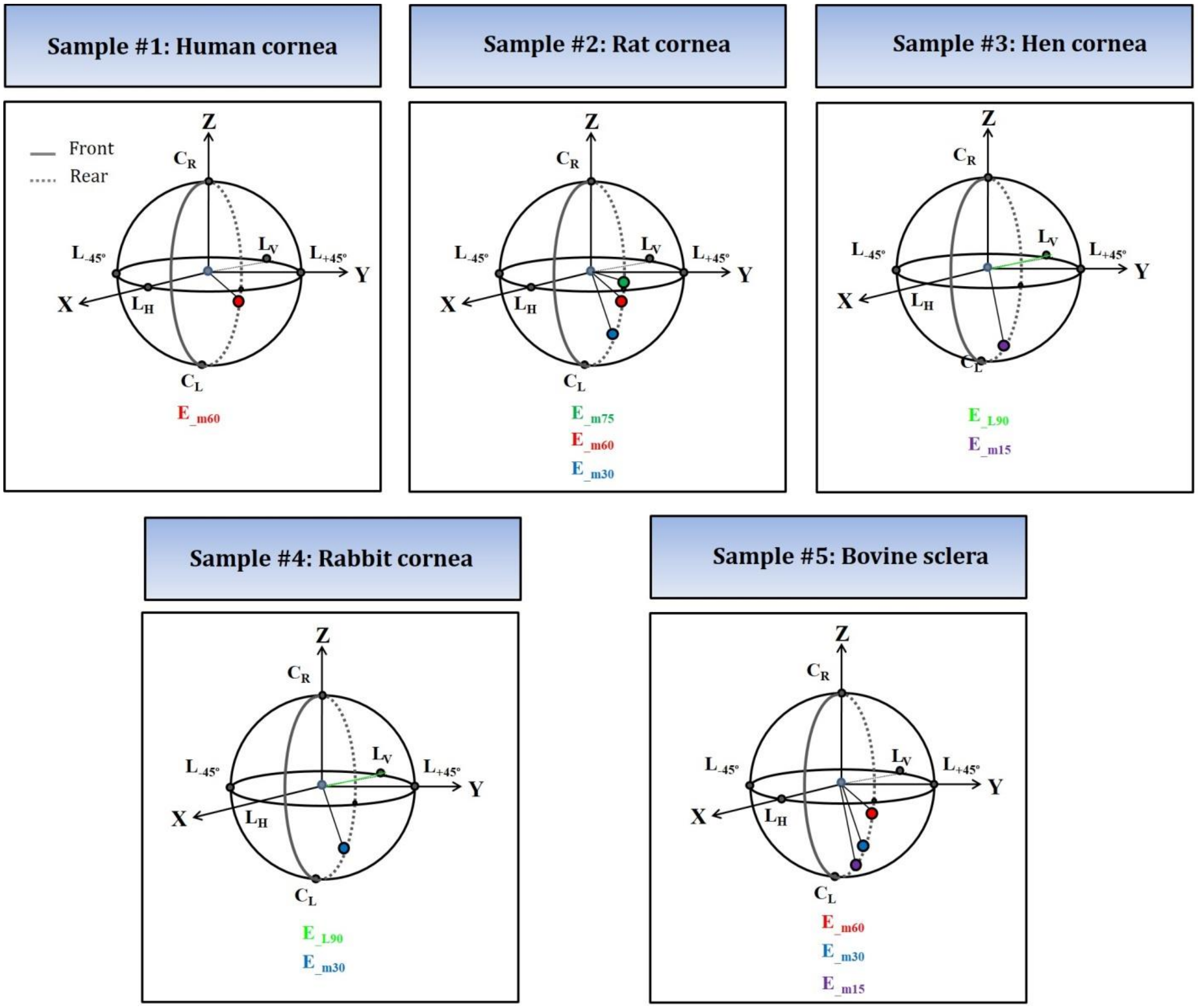

Fig. S1. Positions on the Poincaré sphere of the polarization states associated the PSGH image instances with highest MOS, highest Average Intensity and most voted by the top three NR-IQA methods (see main text for more details).
